# Critical Analysis of Esophageal Multichannel Intraluminal Impedance Monitoring 20 Years Later

**DOI:** 10.5402/2012/903240

**Published:** 2012-10-24

**Authors:** Fernando A. M. Herbella

**Affiliations:** ^1^Department of Surgery, São Paulo Medical School, Federal University of São Paulo, 04021-001 São Paulo, SP, Brazil; ^2^Surgical Gastroenterology, Division of Esophagus and Stomach, Hospital São Paulo, Rua Diogo de Faria 1087 cj 301, 04037-003 São Paulo, SP, Brazil

## Abstract

Multichannel intraluminal impedance (MII) for the evaluation of esophageal diseases was created in 1991 trying to solve previous limitations of esophageal function test. MII-pH is able to determine the physical characteristics of the refluxate (liquid, gas, or mixed) and nonacidic GER. MII-manometry can determine the presence of bolus and its relation with peristalsis. This paper makes a critical analysis of the clinical applications of MII 20 years after its creation. Literature review shows that MII made great contributions for the understanding of esophageal physiology; however, direct clinical applications are few. MII-pH was expected to identify patients with normal acid reflux and abnormal nonacidic reflux. These patients are rarely found off therapy, that is, nonacidic reflux parallels acid reflux. Furthermore, the significance of isolated nonacidic reflux is unclear. Contradictory MII-manometry and conventional manometry findings lack better understanding and clinical implication as well as the real significance of bolus transit.

## 1. Introduction

Esophageal manometry and pH monitoring are ambulatory techniques for detection of gastroesophageal reflux (GER) and esophageal motility disorders that were introduced into clinical practice in the 1970s; however, significant improvement was achieved over the last decades [1], including the development of multichannel intraluminal impedance (MII) in 1991 [2]. These new achievements try to solve previous limitations of esophageal function test, such as the lack of ability to detect bolus transit and nonacid reflux.

 A great enthusiasm came when MII was applied to esophageal physiology. First of all, the expectation that patients with suspected GER and a negative pH monitoring would have the disease objectively diagnosed came into mind. Second, the detection of bolus transport through the esophagus brought hope to the treatment of patients with dysphagia and normal manometry. This initial enthusiasm; however, subsided along time. This paper will focus on the critical analysis of the clinical applications of MII, 20 years after its creation.

## 2. MII Technique

Impedance is the measurement in Ohms of the electrical resistance between 2 points. In simple words, 2 consecutive sensors are in contact with the esophageal mucosa that has specific impedance value, but when the lumen is filled with any substance and this substance bridges these 2 sensors, the equipment will detect this variance. Because of their differential conductivity, gas, liquid, or a mixture of the two can be distinguished independent of the pH of the material. The order in which the sensor detects the material also allows determining the direction of its flow. The passage of liquid substances between the sensors decreases the impedance value. It is detected as a drop in the impedance of more than 50% from the baseline. In contrast, gas has high electrical resistance, leading to an increase in impedance of more than 50% the baseline, or an absolute value >7,000 ohms and mix of gas and liquid will be a combination of both. Return to 50% of the baseline is considered the end of the episode. During deglutition, lumen substances are detected first in the most proximal sensors and then progresses distally. A reflux episode is considered when lumen substances are detected first in distal sensors and then propagates aborally in at least two proximal sensors. Simultaneous detection of an episode of reflux by the pH sensor and by the impedance sensors denotes an acid reflux. Detection of an episode of reflux only by the impedance sensors denotes a non-acid reflux [3, 4].

**Figure 1:**
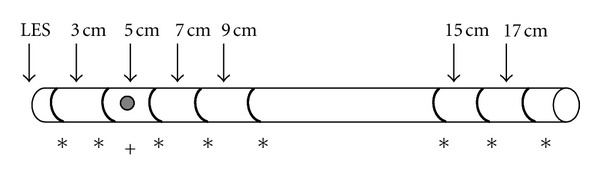
Multichannel Intraluminal Impedance and pH-monitoring catheter. ∗—impedance sensors, +—pH sensors, and LES—lower esophageal sphincter.

 MII-pH is a catheter-based technology. It consists of a catheter comparable to the conventional pH-monitoring where antimony sensors are similarly used to detect pH, but impedance sensors are also displaced in the catheter. The arrangement of the sensors is variable according to the manufacturer; however, the most common placement is shown in Figure 1.

 Baseline measurement of impedance levels may denote mucosal integrity [4, 5] although its clinical significance is still elusive.

## 3. Assessment of Gastroesophageal Reflux

Conventional catheter-based prolonged ambulatory esophageal pH monitoring has been used since the 1970s. Its usefulness in clinical practice and research has been proven and reassured along time [6]. 

 The first attempt to detect GER was accomplished in 1884 by Reichman, who lowered a sponge into the esophagus of a patient with heartburn and showed that it contained acid when retrieved. Over 50 years later, Aylwin in 1953 found acid and pepsin in the esophageal juice—retrieved with the aid of a tube—of patients with esophagitis [1]. The first *in situ* measurement of acid reflux in the esophagus is credited to Tuttle and Grossman in 1958 who used equipment previously described to study gastric pH and combined it with esophageal manometry. They studied different probe positions and pH levels and ultimately concluded that acid drop below 3 at 2 cm proximal to the respiratory inversion point was indicative of acid reflux [7]. Many other studies on the subject followed. Initially, recording machines were not portable, forcing patients to be wired to large equipments, making the procedure an in-patient system [1]. Radiofrequency transmission of pH data is not a modern technology; in fact its development started in the 1950s, and it was almost parallel to the “conventional” catheter technique. However, state-of-the-art technology for catheter-free wireless pH sensors and a new technology to attach the capsule were introduced in 2001 [8].

 Detection of intraluminal esophageal impedance was developed in the early 1990s [2]. The combination of simultaneous detection of esophageal acidity (pH) and impedance-MII was also a landmark in understanding and evaluating GER.

 Gastric and duodenal contents both can reflux into the esophagus and adjacent organs. Gastric hydrochloric acid has long been recognized as harmful to the esophagus [9]. Currently, it is recognized that bile reflux is also noxious to the esophagus [10].

 Detection of non-acidic has been tried for decades. Researchers were motivated by patients with clinical and endoscopic evidence of GER but normal pH monitoring [11, 12], when non-acid reflux was suspected as the etiology of symptoms and mucosal damage in these patients [13, 14] since bile-induced esophagtis en experimental models [15, 16]. Different methods have been developed to detect non-acidic reflux, such as: esophageal intubation and aspiration [17–19], detection of bile in the sputum [20, 21], alkaline pH shift at the pH monitoring [22, 23], and scintigraphy [24]. All described methods did not prove useful due to technical limitations, low sensitivity, nonphysiologic situations, and nonprolonged measurements. Probably the most promising technology was bilirubin monitoring through spectrophotometry (Bilitec 2000). It consists of a portable sensor coupled to a pH sensor in the tip of a catheter. It allows ambulatory measuring similar do conventional pH monitoring [25]. 

 MII-pH goes further than the previous technologies and it is also able to determine the physical characteristics of the refluxate (liquid, gas, or mixed) and non-acidic GER [26, 27]. 

 New concepts became evident only after the advent of MII-pH. For instance, rereflux (or superimposed reflux) is characterized by reflux episodes detected at impedance in a background pH < 4 after the initialization of a primary reflux event [28]. It seems to be associated to patients with severe esophagitis, postprandially, and in the recumbent posture [29]; however, its clinical relevance must be clarified.

Also, the correlation between symptoms and the pH of the refluxate have been studied. It was demonstrated that MII-pH improves the sensibility to correlate symptoms with episodes of reflux, since a significant number of symptoms may occur during non-acid episodes [30]. Various studies showed that heartburn is more commonly experienced in acid reflux [31–33], while regurgitation [31, 32] and cough [33, 34] are symptoms most associated to non-acid reflux.

Different publications studied the effect of antisecretory drugs on the composition of GER. Their results, either studying volunteers [35] or patients [32], showed that the number and duration of reflux episodes were not changed by proton pump inhibitors, only the pH of the refluxate. 

 MMI-pH, different from conventional pH monitoring, can discriminate the physical characteristics of the refluxate (liquid, gas, or mixed). The clinical importance of this characteristic is still elusive. Tutuian et al. [36] have shown that reflux episodes that were associated with symptoms in patients who failed clinical therapy were primarily composed of both gas and liquid. Emerenziani et al. [37] showed that in heartburn patients the risk of reflux perception was significantly higher when gas was present in the refluxate. On the other side, mixed reflux (gas + liquids) comprises half of the episodes of reflux in volunteers [37, 38] and patients with erosive GER, nonerosive GER, and healthy individuals have the same amount of gas containing episodes of reflux [38, 39]. Both surgical [40] and clinical [37] therapy decreases the number of gas-containing reflux episodes; however, it is unclear if symptomatic relieve is linked to this finding since other forms of refluxate are also controlled by these therapies.

## 4. Critical Analysis of MII-pH

Previous methods to detect non-acid reflux (Bilitec, esophageal aspiration, scintigraphy, etc.) were contributory; however, technical limitations precluded widespread use of the technology. Even though, they made great contributions for the understanding of gastroesophageal reflux disease and esophageal physiology. MII-pH is probably the most effective method to detect non-acid reflux. 

 The aforementioned characteristics of the MMI-pH technology also brought great contribution for the understanding of gastroesophageal reflux disease and esophageal physiology. Clinical implications are; however, limited by: (1) studies with controversial results, (2) the rarity of isolated alkaline reflux, and (3) the lack of clinical implication on prognosis, therapeutic decision or postoperative evaluation.

 The prevalence of weak-acidic GER (esophageal pH 4–7 + reflux detected at he MII) is variable in different series. Weakly acidic reflux ranges from 20 to 66% of the episodes of GER in healthy individuals [26, 41, 42]. In GER patients, weakly acidic GER shows no higher percentage—30–70% [43–45]. The reasons for the difference in prevalence of weakly acidic reflux between studies are unclear and require further investigation. Ambulatory impedance pH studies suggest that patients with moderate and severe esophagitis have rates of weakly acidic reflux similar to or slightly greater than healthy controls. Furthermore, distal esophageal exposure to weakly acidic refluxate is similar in esophagitis and nonerosive reflux disease (NERD) patients [46]. Also, the composition of the refluxate must be better clarified. Weakly acidic reflux is more likely to occur early after a meal, both in controls and in patients with reflux disease [47]. Studies using pH-Bilitec recordings have shown that most bile reflux events occur in an acid setting, with esophageal pH below 4 [48]. It is possible that differences in mixing and distribution of postprandial gastric contents might explain the occurrence of either weakly acidic reflux with little or no biliopancreatic secretion [47]. If patients with abnormal weakly acidic reflux and physiologic acid reflux can be classified as refluxers is a question to be answered. Alkaline reflux (pH > 7) is a rare event [26, 41, 42].

 MII-pH has not showed to be useful to predict treatment response. Bredenoord et al. [40] showed that MII parameters for non-acid reflux (symptom association or number of episodes of reflux) are not predictive for response to proton-pump inhibitors in GER patients. Similarly, MII parameters do not predict outcomes after antireflux surgery (fundoplication) [49, 50]. On therapy MII-pH is a common method for the detection of the persistence of GER in patients refractory to pharmacologic therapeutic [51]. Although we honestly believe that for patients with proven GER off therapy, this evaluation is unnecessary. 

 MII-pH has not shown to be useful in the postoperative period of antireflux operation (fundoplication). Arnold et al. [52] showed that in asymptomatic patients with a negative pH monitoring, the rate of false positive MII-pH of 50% renders the test clinically irrelevant. This may explain findings such as a lack of decrease in nonacid reflux episodes after endoscopic fundoplication as it is not selective to the type of refluxate [53].

 Further considerations are necessary. Catheter-based esophageal function tests are nonphysiologic methods. One of the reasons appointed to a false-negative pH monitoring is alteration in normal life style, food intake, and hypersalivation due to the presence of the transnasal catheter. Wireless catheter-free implantable capsule-based pH-monitoring was created to prevent the discomfort associated to the presence of the esophagonasal catheter [54, 55]. Unfortunately, untoward effects and limitations are frequent, most of them related to the fixation method that pins the capsule to the esophageal mucosa. Thus, problems reported are: (1) the capsule fails to deploy and attach to the esophageal mucosa in a significant percentage of patients (ranging from 0.5 to 20% [56–60]); (2) the presence of the capsule in the esophagus may cause symptoms. The majority of patients experience foreign body sensation, especially with swallow and dysphagia [56, 60, 61]. Chest pain occurs in 33–50% of patients previously free of this symptom [58, 59]. The pain may be severe in 1 to 9% of the patients and removal of the capsule may be occasionally necessary in 2 to 5% of the patients, from 1 hour up to 5 days after the procedure [61]. Nausea was also reported in 6% of the studies [58]; (3) in a significant number of studies (2 to 12% [56, 57, 60]) data cannot be completely retrieved. Reasons for incomplete data retrieval are unexplainable failure of the device (either the capsule or the receiver), interference due to other wireless devices, permanence of the receiver to far from the patient, and detachment of the capsule. The capsule is designed to detach in 3–7 days and be expelled in the stool. Premature detachment of the capsule can occur in 2 to 5.5% of the cases [56, 57, 59, 60]; (4) in the opposite direction, nondetachment of the capsule after 15 days requiring endoscopic removal was reported [61]; (5) the number of reflux episodes are consistently lower when Bravo capsule is compared to conventional pH monitoring [59]. This is credited to the lower sampling rate (every 6.25 seconds, compared to 4 per second) of the Bravo system [62], fixed position of the wireless capsule that prevents it to dip inside the stomach during swallows [63], and inaccuracy in calibration of the capsule [64]; and (6) a precise positioning based on the LES can be accomplished using Bravo system if manometry is done previously and transnasal route is used to place the capsule. However, the squamous-columnar transition, a variable anatomical position, is utilized as landmark if the transoral route is used. Currently, there is no system able to measure nonacid reflux through a wireless capsule. Other point is a prolonged (more than 1 day) measurement of GER. Different studies showed that increasing the period of analysis increases the sensitivity for GER detection [55, 65, 66].

## 5. Assessment of Esophageal Motility

The same technology for MII-pH has been used in combination with esophageal manometry. MII-manometry can determine the presence of bolus and its relation with peristalsis. Analysis of MII-manometry is very similar to the analysis of MII-pH. Transit of the bolus induces the same pattern of impedance measurement. 

**Figure 2:**
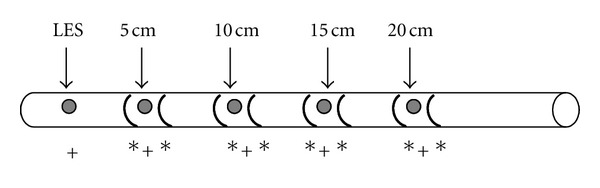
Multichannel intraluminal impedance and esophageal manometry catheter. ∗—impedance sensors, +—manometry sensors, and LES—lower esophageal sphincter.

 Similarly to MII-pH, impedance sensors are also displaced along a conventional manometry catheter. Both water perfused [32] and solid state [28] catheters can be used, although the former is more common. The arrangement of the sensors is variable according to the manufacturer; however, the most common format is shown in Figure 2.

**Figure 3:**
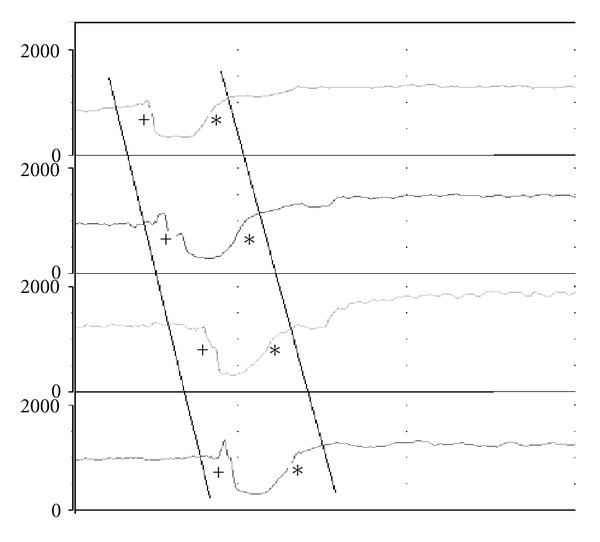
Impedance parameters in the multichannel intraluminal impedance and esophageal manometry. Bolus clearance is complete if bolus is detected at the most proximal sensor and it “exits” all sensors in sequence, as shown in the figure. Bolus transit time is the period of time between the first and the last detection of the bolus, in the most proximal and in the most distal sensor, respectively. +—begin of bolus detection and ∗—end of bolus detection.

 Analysis of MII-EM tracings is very similar to the analysis of MII-pH. Transit of the bolus induces the same pattern of impedance measurement. Usually, 10 viscous swallows, not only 10 liquid swallows, are usually added to the analysis [67] allowing higher detection of esophageal function defects compared to liquid swallows [68]. Obviously, the addition of viscous swallows can be integrated to the conventional manometry as well [69]. More recently, solid swallows have also been added to MII [70]. Multiple parameters can be recorded; nevertheless bolus clearance and transit time are simple but informative parameters (Figure 3). This technology allows (1) monitoring of bolus transport patterns, (2) calculation of bolus transit parameters, (3) evaluation of bolus clearance, (4) monitoring of swallow associated events such as air movement and reflux, and (5) investigation of the relationships between bolus transit and LES relaxation [71].

 Tutuian and Castell [72] studying 350 patients with a wide range of esophageal diseases reported that complete bolus transit detected with the impedance was identified in 96% of manometric normal swallows, 33% of ineffective, and 53% of simultaneous waves considering liquids swallows. Furthermore, distal esophageal amplitude was higher in patients with complete bolus clearance, as expected. 

Different motility disorders have been studied by MII-manometry. Published studies showed that patients with achalasia and scleroderma have always abnormal bolus transit [72–74]. Almost half of patients with ineffective esophageal motility and diffuse esophageal spasm have normal bolus transit, while almost all patients with normal esophageal manometry, nutcracker esophagus, poor relaxing LES, hypertensive LES, and hypotensive LES have normal bolus transit [72, 74]. The analysis of these results according to subgroups of diseases, not only reinforces some previous concepts but also changes some of them. Esophageal motility abnormalities can be classified as transit defect or pressure defect [75]. Some conclusions are: (a) achalasia and scleroderma are associated to manometric abnormalities and abnormal bolus transit, as expect; (b) isolated sphincter abnormalities do not affect bolus transit; (c) ineffective esophageal motility may have a normal bolus clearance; (d) diffuse esophageal spasm may have a normal bolus clearance; (f) nutcracker esophagus is a disease of abnormal pressures, not abnormal bolus transit; and (g) abnormal bolus clearance can be seen in a small number of patients with normal manometry.

MII role in the workup for belching disorders and rumination seems promising [76].

## 6. Critical Analysis of MII-Manometry

Similarly to MII-pH, MII-manometry made great contributions for the understanding of esophageal physiology; however, direct clinical application is jeopardized by 3 points: (1) different MII findings are found in the same series of patients; (2) abnormal MII findings did not prove a real value in changing current treatment options; (3) MII-manometry does not predict treatment outcomes; and (4) the significance of discrepant MII and manometry results is elusive.

 A percentage of altered and normal MII has been reported in healthy volunteers [55], patients with dysphagia and without dysphagia [77, 78], and patients in the postoperative of Nissen fundoplication with and without dysphagia [79]. Bogte et al. [80] affirmed that stasis of both liquid and solid boluses occurs frequently in patients and in controls and can be regarded as physiological!

**Figure 4:**
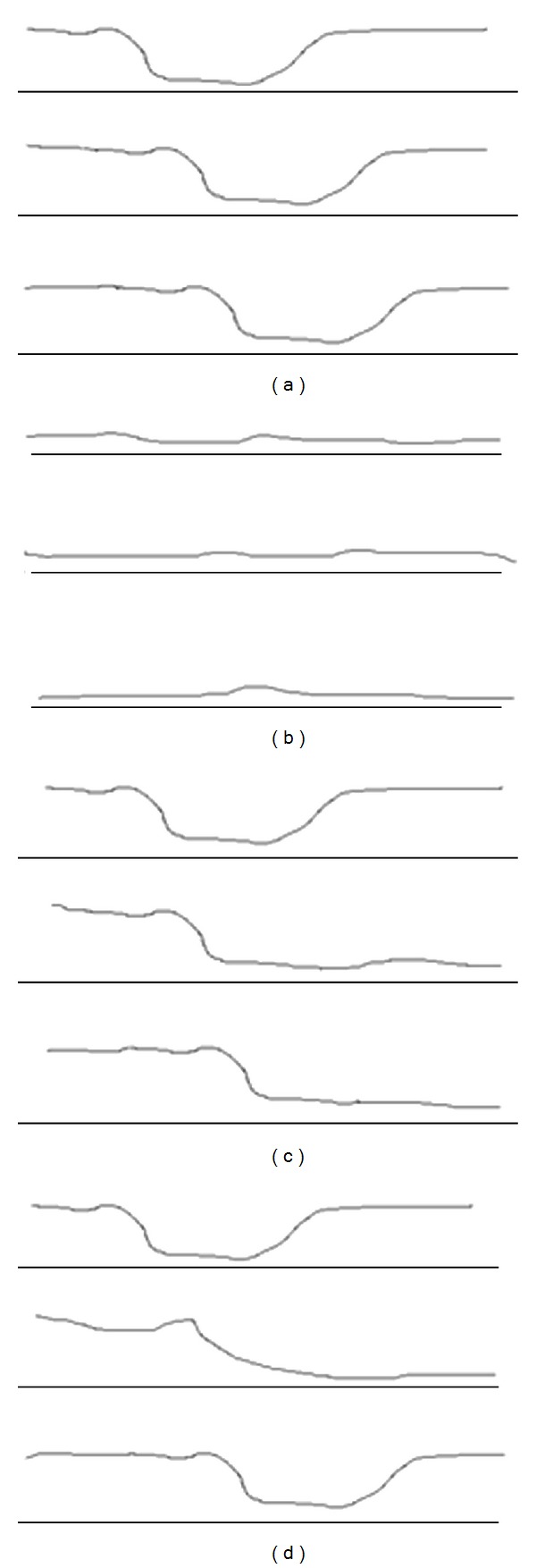
Multichannel intraluminal impedance (MII) patterns for bolus transport. (a) Normal bolus transport. A decrease in basal impedance measurement and subsequent return to basal levels is noticed in all channels (proximal, medium, and distal). (b) Abnormal bolus transport. All channels display a low basal impedance level unchanged by swallow. This may be explained by fluid repletion of the esophagus? It is a common pattern in achalasia patients. (c) Abnormal bolus transport. The proximal channel shows a normal bolus propagation. More distal channels display a retention of the bolus distally. This may be explained by outflow resistance at the esophagogastric junction. (d) Abnormal bolus transport. Retention of the bolus is noticed in the midesophagus. This may be explained by segmental aperistalsis.

 Classically, esophageal motility disorders have been classified, named, and treated based on manometric characteristics [81]. The recent advent of high-resolution manometry allowed the development of a different classification [82] but again with direct clinical implications for treatment [83, 84]. Moreover, MII have been recently coupled with high-resolution manometry [85–89], interestingly; however, MII proved to validate high-resolution manometry ability to detect bolus transport and not the opposite [89, 90]. No MII-specific patterns were identified in order to create dysmotility classifications. Also, the lack of correlation between symptom (dysphagia) and abnormal bolus transit increases the confusion to understand MII-manometry findings [91, 92]. Furthermore, low baseline impedance levels, air entrapment and erratic liquids movement in the esophagus limit the application of MII in achalasia and other motility disorders with serious impairment of esophageal clearance [3, 6, 93] (Figure 4). 

 MII-manometry did not show to be useful to predict dysphagia after antireflux operations [78]. Impaired flow through the esophagogastric junction may lead to dysphagia after fundoplication, however, this finding correlates with conventional manometric findings [94]. 

 The combination of impedance and esophageal manometry allowed the identification of 4 patterns of swallows: (1) peristaltic waves (based on manometry) and bolus cleared (based on MII); (2) peristaltic waves without bolus clearance; (3) nonperistaltic or ineffective waves and bolus cleared; and (4) nonperistaltic or ineffective waves without bolus clearance. It is intuitive to understand and explain the concordance of MII and manometry findings represented by previous items (1) and (4). The understanding of items (3) and (4) boggles the mind to explain how bolus transit and muscular contraction are disconnected. 

## 7. Conclusions

MII made great contributions for the understanding of esophageal physiology; however, direct clinical applications are few.

 MII-pH was expected to identify patients with normal acid reflux and abnormal nonacidic reflux. Unfortunately, these patients are rarely found off therapy, that is, nonacidic reflux parallels acid reflux [95]; and the significance of isolated nonacidic reflux is unclear [1]. Repeating words by Sifrim and Zerbib [96]: “Combined pH-impedance has little added value in patients ‘off' therapy and virtually no outcome data exist to determine the optimal pH-impedance parameters.”

 The significance of bolus transit is elusive. MII-manometry findings that contradicts manometry lacks better understanding and clinical implication. 

 Future technologies may fill these clinical expectancies. Molecular imprinting technology with biosensors to detect bile [97], an implantable, wireless, and batteryless impedance sensor capsule that infers impedance based on a direct measurement of capacitance and receives energy from an external source [98], and intraluminal miniultrasound [99] are examples in development.
